# Conjugated STING agonists

**DOI:** 10.1016/j.omtn.2025.102530

**Published:** 2025-03-31

**Authors:** Shuhao Qu, Hong Dai

**Affiliations:** 1 School of Veterinary Medicine, Henan University of Animal Husbandry and Economy, Zhengzhou 450046, China; 2 Department of Chemistry, The Hong Kong University of Science and Technology, Clear Water Bay, Kowloon, Hong Kong SAR, China

**Keywords:** MT: Delivery Strategies, stimulator of interferon genes, STING, STING agonists, conjugated, CDNs, clinical trials

## Abstract

An innate immune system is the first line of defense and prevents the host from infection and attacks the invading pathogens. Stimulator of interferon genes (STING) plays a vital role in the innate immune system. STING activation by STING agonists leads to phosphorylation of TANK-binding kinase 1 (TBK1) and interferon regulatory factor 3 (IRF3) with the release of type I interferons and proinflammatory cytokines, further promoting the adaptive immune response and activating T cells by increased antigen presentation. Natural STING agonist cyclic dinucleotides (CDNs) encounter many defects such as high polarity by negative charges, low stability and circulative half-life, off-target systemic toxicity, and low response efficacy in clinical trials. To overcome these challenges, massive efforts have addressed chemical modifications of CDNs, development of non-CDN STING agonists, and delivery of these STING agonists either by conjugation or liposomes/nanoparticles. Considering there have been a great number of reports regarding nanosystem-aided delivery, here, we examine the development of STING agonists, especially for non-CDNs and their delivery specifically by conjugation strategy, with a focus on the STING agonists in clinical trials and current challenges of their potential in cancer immunotherapy.

## Graphical abstract

**Figure:**
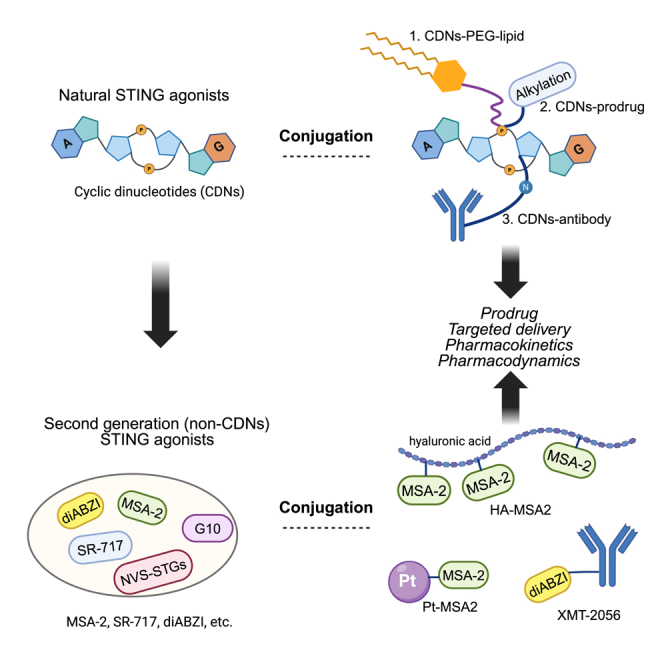

## Teaser

While nanoparticle-mediated delivery has dominated STING agonist research, this review highlights the emerging paradigm of STING agonists especially for non-cyclic dinucleotides (non-CDNs) engineered through covalent conjugation strategies, with a special focus on these STING agonists in clinical trials.

## Introduction

The cellular innate immune system is vital to protect the host from pathogen infection. For the rapid detection of pathogens, cells have evolved various pattern-recognition receptors (PRRs). Cyclic guanosine monophosphate-adenosine monophosphate (cGAMP) synthase (cGAS), one of the most important cytoplasmic DNA sensors, was discovered in 2013.^1^ Following the detection of pathogen DNA or self-DNA in the cytoplasm, cGAS is capable of catalyzing the production of 2′,5′-3′,5′-cyclic guanosine monophosphate-adenosine monophosphate (2′3′-cGAMP). 2′3′-cGAMP in turn binds and activates adaptor protein stimulator of interferon (IFN) genes (STING), thus leading to innate immune signaling cascade, including the release of type I IFNs and other cytokines.^2^

Cyclic dinucleotides (CDNs) are important second messengers in bacteria, and they also serve as natural STING agonists in mammal innate immune systems. In terms of the druggable development in cancer immunotherapy, CDNs face various challenges brought on by their physiochemical properties such as high polarity, short plasma half-life, and poor cellular targeting and membrane permeability.^3^ To overcome these challenges, massive efforts have been focused on the structural modifications and targeted delivery of CDNs.^4^ In addition, non-CDN-type STING agonists have also sprung up in the past decade, and some could have better pharmacokinetics and pharmacodynamics in animal models than natural CDNs.^5^

This review aims to provide the historical development of STING agonists, especially for non-CDNs, and recent strategies for their druggable development, with a focus on the chemical modifications/conjugations to STING agonists in clinical trials.

## PRRs

Recognition and response to pathogenic microbial infection are essential for cell survival. The innate immune system, utilized as the first line of defense against pathogens and harmful substances by the host defense system, generally uses receptors with fixed specificity to recognize conserved features of microorganisms, often called germline-encoded PRRs.^6^^,^^7^ In most cases, the microbial features recognized by innate receptors are common to a wide range of microbial types and are molecules that are clearly unrelated to the host. Nucleic acids are an exception, because nucleic acids are shared by pathogens and hosts, and these nucleic acid receptors may respond to their ligands. Despite this, the innate immune system uses a variety of innate immune receptors that recognize nucleic acids owing to the very reliable microbial recognition that does not easily mutate to avoid receptor recognition. For example, innate immune detection of viruses relies almost entirely on recognizing viral nucleic acids.^8^ Most PRRs in vertebrates are divided into the following categories including Toll-like receptors (TLRs), nucleotide oligomerization domain-like receptors, retinoic acid-inducible gene-I (RIG-I)-like receptors, C-type lectin receptors, absent in melanoma-2 (AIM2)-like receptors,^9^ and cGAMP cGAS.^10^

PPRs that recognize nucleic acids can be divided into two categories based on the different localization of nucleic acids in the cell. The TLR family, such as TLR3, TLR7, and TLR9, monitors the presence of double-stranded RNA (dsRNA) and single-stranded RNA in the endolysosomal compartment. They are capable of binding to ligands released during microbial degradation and transmit signals, thereby detecting pathogenic microorganisms before cells become infected. After pathogen nucleic acid enters the cytoplasm, cells need another type of cytoplasmic receptor to monitor. For example, RIG-I can recognize short dsRNA and 5′-triphosphorylated RNA, while melanoma differentiation-associated protein 5 recognizes long dsRNA.

Despite the various cytoplasmic receptors, the detection of cytoplasmic DNA derived from either non-self-DNA or self-DNA can initiate the activation of innate immune system mostly through the induction of the release of active factors such as type I IFN^11^ after the recognition by PPRs such as DNA-dependent activator of IFN-regulatory factors,^12^ IFN-inducible protein (AIM2),^13^ nuclear IFN-inducible protein 16,^14^ IFN-inducible protein,^15^ myeloid cell nuclear differentiation antigen,^16^ leucine-rich repeat binding FLII interacting protein 1,^17^ and others. However, these PRRs are not universal because they are cell type or DNA sequence specific.

## cGAS and STING

**Figure 1:**
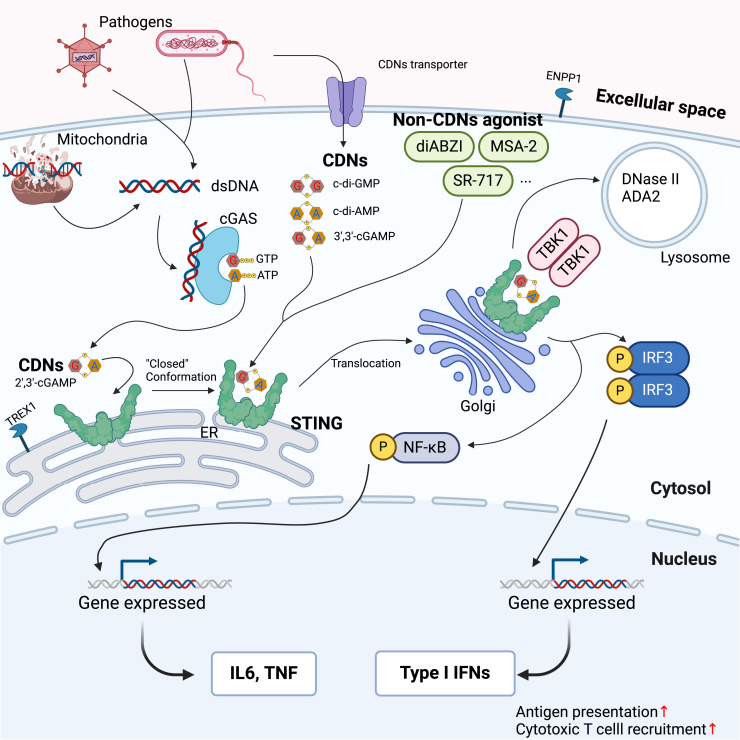
Overview of STING signaling pathway DNA from damaged mitochondria or pathogens can be recognized by cGAS, and cGAS subsequently catalyzes the formation of 2′,3′-cGAMP. The resulting 2′,3′-cGAMP binds to STING with “closed” conformation along with translocation. The phosphorylation of transcription factors IRF3 and NF-κB leads to the upregulation of type I IFNs, pro-inflammatory cytokines, and chemokines. CDNs derived from pathogens such as c-di-GMP, c-di-AMP, and 3′,3′-cGAMP and non-CDNs such as SR-717, MSA-2, and diABZI can also trigger the STING signaling pathway.^1^^,^^18^ Safeguards that limit cGAS-STING activation^26^ include the nucleases TREX1 at the ER and DNase II or ADA2 in the lysosome, the export of cGAMP and subsequent degradation of cGAMP by ENPP1, and the degradation of STING in the lysosomal compartment.

In 2013, one of the most important cytoplasmic DNA sensors was discovered to be cGAS, which is also called Mab-21 domain-containing protein 1. cGAS is an innate immune sensor of cytoplasmic DNA,^18^ HIV, and other retroviruses.^1^ The activation of cGAS by cytoplasmic DNA is strongly DNA length dependent. DNA ligands <20 bp are capable of binding cGAS and forming a 2:2 complex, but they fail to activate cGAS, possibly owing to the complex instability.^19^ Longer DNA, >20 bp, can be recognized to form stable cGAS_2n_-DNA_2_ ladders (*n* ≥ 2) and robustly activate cGAS.^20^ The binding between cGAS and DNA ligands can lead to reassembling the activated conformation^21^ and then catalyzing the synthesis of cGAMP^22^ from adenosine triphosphate (ATP) and guanosine triphosphate (GTP), which was demonstrated to own G(2′,5′)pA and A(3′,5′)pG phosphodiester linkages, designated as c[G(2′,5′)pA(3′,5′)p], or 2′,3′-cGAMP.^23^^,^^24^ The resulting 2′,3′-cGAMP in turn activates STING, followed by the translocation of the complex from the endoplasmic reticulum (ER) to the Golgi apparatus,^25^ leading to the activation of TANK-binding kinase 1 (TBK1) to promote the phosphorylation and dimerization of IFN regulatory factor 3 (IRF3). It may also activate I B kinase to promote the phosphorylation of nuclear factor κB (NF-κB). Phosphorylated IRF3 and NF-κB enter the nucleus to induce the expression of IFN and inflammatory cytokines and chemokines, which play a vital role in immunostimulatory functions by promoting the priming and activation of T cells, dendritic cells (DCs), and natural killer cells, ultimately inducing an immune response (Figure 1).^27^^,^^28^ Despite the activation of potent immune response, STING also plays an indispensable role in chronic inflammation and functional decline during aging,^29^ as well as in systemic and organ-specific diseases.^30^

STING is a small-size (∼80 kDa for a dimer) membrane protein predominantly located in the ER, which has 379 amino acid sequences in human cells and 378 amino acids sequences in murine cells.^31^ It consists of a short N-terminal cytosolic segment and four transmembrane helices encompassing approximately 130 amino acids, a cytosolic ligand-binding domain (LBD) and a C-terminal tail that is responsible for binding TBK1, taking up the final 250 amino acids.^32^^,^^33^ The LBD serves as the functional dimer that recognize dsDNA and second messenger CDNs, including cyclic di-guanylate monophosphate (c-di-GMP),^34^ cyclic di-adenylate monophosphate (c-di-AMP),^35^ 3′,3′-cyclic guanosine monophosphate-adenosine monophosphate (3′,3′-cGAMP),^36^ 3′,2′-cyclic guanosine monophosphate-adenosine monophosphate (3′,2′-cGAMP), and 2′,3′-cGAMP.^23^^,^^24^ STING is polymorphic in humans and consists of mainly five variants: R232 (58%, generally referred to the wild type), HAQ (20%), H232 (13%), AQ (7%), and Q (2%). The binding affinity of intrinsic 2′,3′-cGAMP to human STING is much higher than the other CDNs, which could be attributed to its less entropic and enthalpic costs when binding to STING and the importance of position 232, which also contributes to the ligand specificity.^37^^,^^38^ A cryoelectron microscopy study of full-length STING in both apo and 2′,3′-cGAMP-bound states suggests that the transmembrane and cytoplasmic regions interact to create a dimeric assembly, with 2′,3′-cGAMP-induced closure of the LBD causing a 180° rotation relative to the transmembrane domain (TMD). This rotation triggers a conformational change that facilitates the formation of the STING tetramer and higher-order oligomers through lateral packing.^39^

## Natural CDN STING agonists

**Figure 2:**
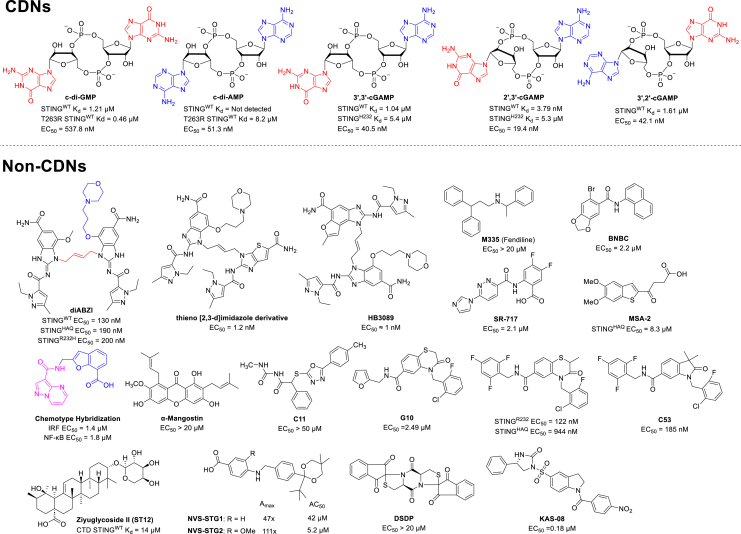
Structures of CDNs and representative non-CDN STING agonists The binding affinity with human STING (mainly by isothermal titration calorimetry), and EC_50_ values of IFN induction are also listed.^40^^,^^41^^,^^42^^,^^43^

To date, five natural CDNs have been reported (Figure 2): c-di-GMP, c-di-AMP, 3′,3′-cGAMP, 2′,3′-cGAMP, and 3′,2′-cGAMP.

### c-di-GMP

c-di-GMP is a ubiquitous second messenger molecule in bacteria that is involved in regulating a variety of bacterial physiological functions, including bacterial communication, cell differentiation, biofilm formation, and cell morphology changes.^44^ In 1987, the Benziman group discovered that a guanylate oligonucleotide-like substance activated cellulose synthase by allosteric activation when studying the cellulose biosynthesis pathway of glucose xylinum. Later, it was confirmed that the oligonucleotide was c-di-GMP. c-di-GMP is usually synthesized by proteins containing the GGDEF domain, and phosphodiesterases (PDEs) containing the EAL domain are generally enzymes that degrade c-di-GMP in bacteria.^45^

Currently, a variety of c-di-GMP receptor proteins have been discovered. In 2006, Dorit Amikam and Michael Y. Galperin used bioinformatics to predict the first c-di-GMP binding protein, the PilZ domain protein.^46^ In the same year, Gomelsky et al. confirmed that this receptor protein, the PilZ domain protein YcgR purified from *Escherichia coli*, can bind to c-di-GMP (K_D_ = 0.84 ± 0.16 μM). A conserved sequence RxxxR20–30(D/N)x(S/A)xxG (x is an amino acid) contained in the PilZ domain is the key site for binding to c-di-GMP.^47^ Another type of c-di-GMP receptor is a protein containing a GGDEF or EAL domain, such as the EAL domain of *Pseudomonas aeruginosa* FimX, which can bind to c-di-GMP to regulate its twitching motility.^48^ In addition, there are some c-di-GMP receptor proteins that do not belong to the above two types, such as FleQ of *P. aeruginosa*^49^ and VpsR of *Vibrio cholerae*.^44^

Another type of c-di-GMP receptor is a riboswitch. Riboswitches are RNA aptamers, non-coding regions of mRNA that can adopt special secondary structures to bind small-molecule ligands. After the riboswitch binds to the small-molecule ligand, the secondary structure of the mRNA changes, leading to changes in transcription, stability, or downstream gene translation. Breaker et al. discovered the first riboswitch, GEMM, for c-di-GMP,^50^ and in 2010 discovered a second type of riboswitch that is closely related to c-di-GMP-induced RNA splicing.^51^ Riboswitches bind to c-di-GMP with very high affinity (nanomolar level) and can control the gene expression of many basic cellular physiological processes, such as virulence gene expression, pili formation, and flagellar organelle generation.

### c-di-AMP

c-di-AMP is another bacterial second messenger discovered in 2008. When studying the mechanism of DNA damage checkpoint activation, Karl-Peter Hopfner’s group^52^ found that after DNA double-strand breakage, DisA (DisA is a protein that controls the sporulation checkpoint of *Bacillus subtilis* in response to DNA breakage) forms a large octamer with two binding domains, one of which has adenylate cyclase activity and binds to a nucleotide that has never been reported before, c-di-AMP. In 2013, Zheng-Guo He’s group^53^ first confirmed the receptor protein Ms5346 for c-di-AMP, which is a TetR family regulator of mycobacteria. In the same year, Vincent T. Lee’s group^54^ discovered several conserved receptor proteins for c-di-AMP, such as potassium transporter-gating component KtrA and cation/proton antiporter CpaA. These proteins have a common feature, the RCK_C (K + conductance regulator) domain, which is the key site for binding to c-di-AMP. c-di-AMP is synthesized by diadenylic acid cyclases and degraded into a linear pApA structure by PDEs contained in GGDEF domain proteins.^55^

### 3′,3′-cGAMP

3′,3′-cGAMP is another CDN second messenger molecule discovered in bacteria in 2012. J.J. Mekalanos’s group identified a class of small RNAs encoded by the toxin-coregulated pilus (TCP) island when studying the role of Vibrio seventh epidemic island-1 (VSP-1) in the pathogenesis of cholera. This class of RNAs can reduce the expression of the transcription factor VspR. VspR regulates the expression of several VSP-1 genes, including a class of genes encoding a novel dinucleotide cyclase (DncV), which can preferentially synthesize a new class of CDN molecules, namely 3′,3′-cGAMP. The above pathway defines VSP-1 as a pathogenicity island in *V. cholerae*, which regulates host adaptability by producing the CDN 3′,3′-cGAMP, which is of great significance to the enhancement of infectivity and reduction of chemotaxis of *V. cholerae*.^36^

In 2015, Xiaodong Su’s research group reported three PDEs (V-cGAP1/2/3) in *V. cholerae* that specifically degrade 3′,3′-cGAMP. Among them, V-cGAPs (V-cGAP1/2/3) are proteins containing HD-GYP fragments that can only specifically degrade 3′,3′-cGAMP but no other types of CDNs. During the degradation process, V-cGAPs first degrade 3′,3′-cGAMP into linear 5′-pApG, and then V-cGAP1 dephosphorylates 5′-pApG to generate 5′-ApG. V-cGAP1 participates in the two-step degradation process, but plays a different role in each step and has high specificity.^56^ Shanahan’s group reported that the C92U mutation in the ligand binding pocket of the type I c-di-GMP riboswitch enabled the riboswitch to bind 3′,3′-cGAMP.^57^ In 2015, Hammond et al. discovered a new type of bacterium that can synthesize 3′,3′-cGAMP, *Geobacter metalloreductus*, which uses the GEMM-I subtype riboswitch (GEMM-Ib—i.e., genes that control the environment, cell membrane, and movement) as a specific receptor for 3′,3′-cGAMP.^58^

### 2′,3′-cGAMP

In 2013, Zhijian J. Chen et al.^22^ studied the specific mechanism of cytoplasmic DNA in inducing IFN release in natural immunity and found that cytoplasmic DNA can induce the production of a new type of CDN molecule, 2′,3′-cGAMP, which directly or indirectly activates IRF3 to induce the release of IFN-β. While studying how cells sense cytoplasmic DNA, the research group discovered cGAS. cGAS can sense cytoplasmic DNA and bind to it, catalyzing ATP and GTP to form 2′,3′-cGAMP, which exerts an immune response through a signal cascade.^18^ Later, the research group also confirmed that cGAS is also a cytoplasmic receptor for retroviruses such as HIV, simian immunodeficiency virus, and murine leukemia virus.^1^

In 2014, a glycoprotein, ectonucleotide pyrophosphatase/phosphodiesterase (ENPP1), was discovered to be located on the cell membrane and ER, and this enzyme can degrade 2′,3′-cGAMP into ATP and GTP (K_cat_ = 4 s^−1^, K_m_ = 15 μM).^59^

### 3′,2′-cGAMP

One recent study identified another type of CDN, 3′,2′-cGAMP, in model organism *Drosophila melanogaster*.^60^ One of the cGAS-like receptors (cGLRs), *Dm*-cGLR1, is capable of recognizing dsRNA and synthesizing one novel second messenger cG[3′–5′]pA[2′–5′]p (3′,2′-cGAMP). *Drosophila* STING exhibits decreased affinity to 2′,3′-cGAMP rather than 3′,2′-cGAMP, which is because of R229 (equal to position 232 in human STING) repositioning and the N159 interaction with adenosine 3′-OH in 3′,2′-cGAMP, similar to human STING S162 with the guanosine 3′-OH in 2′,3′-cGAMP. In addition, this isomeric switch in the specificity of phosphodiester linkage makes 3′,2′-cGAMP resistant to cleavage by poxin, enabling *Drosophila* capability from viral immune evasion. *Dm*-cGLR2 can also guide the production of a combination of 2′,3′-cGAMP and 3′,2′-cGAMP and orchestrate an STING- and NF-κB-dependent antiviral immune response, but the activator is not yet identified.^61^ 3′,2′-cGAMP can be activated and synthesized by CdnG, which in turn activates both *Asticcacaulis* sp. Cap5 and *Lactococcus lactis* Cap5 for DNA degradation through cyclic-oligonucleotide-based antiphage signaling systems.^62^ The transcriptional landscape of 3′,2′-cGAMP signaling in mammals shows that this signaling preferentially induces many STING-dependent genes involved in transcription and nucleosome positioning and assembly in the nucleus compared to 2′,3′-cGAMP.^63^

## Non-CDN STING agonists

Structurally, natural STING agonists, CDNs, are confronted with two main defects, including poor cellular membrane permeability owing to the two negatively charged ions under physiological conditions and instability under certain PDEs. These inherent characteristics largely hinder their therapeutic applications. Therefore, the development of a novel class of non-CDN STING agonists has emerged, especially in the past decade (Figure 2; Table S1).^40^

Prior to the 2′,3′-cGAMP discovery, some other types of STING agonists have been reported, including 5,6-dimethylxanthenone-4-acetic acid (DMXAA), flavone acetic acid (FAA), and 10-carboxymethyl-9-acridanone (CMA). DMXAA and FAA are flavonoid derivatives and have shown impressive activity against solid tumors in mouse models, but all of them failed in clinical trials, which is thought to be from a lack of binding affinity toward human STING.^64^ CMA was discovered in the early 1970s and its antiviral action was demonstrated to be STING dependent recently. CMA displays extraordinary activity in IRF3 phosphorylation in the murine system, but it also fails to induce immune response in human cells.^65^ It is noteworthy that these STING agonists can only stimulate mouse STING instead of human STING.

### Dispiro diketopiperzine, BNBC, and C11

The first example of a non-CDN human STING agonist was reported in 2017 via the HepAD38-derived reporter cell line-based high-throughput screening (HTS). This reporter cell line is capable of expressing firefly luciferase (LUC) in response to the activation of the cGAS-STING pathway. A dispiro diketopiperzine (DSDP) compound was identified from a library containing 16,000 compounds, which could induce STING-dependent release of IFN-dominant cytokine response in human skin fibroblasts and peripheral blood mononuclear cells.^66^ It is noteworthy that this DSDP treatment only induces a comparable expression of IFN-β but diminished expression of interleukin-29 (IL-29), tumor necrosis factor α, and IL-6 at the 50-μM level, while 2′,3′-cGAMP requires only around 10 μg/mL (∼14 μM); in addition, DSDP is more likely to enter cells with a better transmembrane efficiency. Two years later, this group reported another STING agonist called 6-bromo-*N*-(naphthalen-1-yl)benzo[d][1,3]dioxole-5-carboxamide (BNBC) by HTS using the HepG2/STING/IFN-stimulated gene (ISG)54Luc cell. Importantly, BNBC can activate only human STING instead of murine STING, induce cytokine release such as IFN-β and IL-29 (half-maximal effective concentration [EC_50_] around the same as DSDP), and promote the DC maturation necessary for adaptive immune system.^67^

In 2018, another novel compound, *N*-(methylcarbamoyl)-2-[5-(4-methylphenyl)-1,3,4-oxadiazol-2-yl]sulfanyl-2-phenylacetamide (C11), was identified through HTS from ∼52,000 molecules, with the platform construction of telomerase-transduced foreskin fibroblasts stably transduced with green fluorescent protein and LUC reporter proteins. C11 is able to induce antiviral type I IFN in human fibroblasts and myeloid-derived MM6 cells but not in murine RAW264.7 monocytic cells and THP-1 cells. C11-induced antiviral response requires STING participation but not through direct binding like 2′,3′-cGAMP.^68^

#### α-Mangostin

α-Mangostin is a dietary xanthone isolated from mangosteen and has already shown antitumor and antiviral activities. Given that α-mangostin and DMXAA share the same xanthone skeleton, it has been correlated with the cGAS-STING signaling pathway and identified as a human STING agonist, as well as a weaker murine STING agonist. α-Mangostin at 25 μM could induce comparable release of IFN-β in comparison to 8 μg/mL 2′,3′-cGAMP in THP-1 cells.^69^

#### Amidobenzimidazole

The first comprehensive development of a non-CDN STING agonist was in 2018 (GlaxoSmithKline).^70^ Unlike previous reporter cell line-based HTS, competitive binding of ^3^H-cGAMP targeted at human STING was utilized for the HTS platform, leading to the identification of a representative amidobenzimidazole (ABZI) with modest half-maximal inhibitory concentration (IC_50_) at 14 ± 2 μM. Inspired by the co-crystal structure of ABZI and STING, a linker to replace the N1 moiety was used to form dimerized compound diABZI, which shows a 1,000-fold increase of IC_50_ at 20 ± 0.8 nM. This molecule diABZI has a K_d_ value of ∼1.6 nM to human STING versus 3.79 nM^71^ for 2′,3′-cGAMP and can induce dose-dependent secretion of IFN-β with an EC_50_ of 3.1 ± 0.6 μM versus 53.9 ± 5 μM for 2′,3′-cGAMP, despite a relatively low induction of the maximum amount. It is also noteworthy that the binding after diABZI maintains the open conformation of STING, unlike 2′,3′-cGAMP. Further compound structural optimization could achieve a minimal EC_50_ of 130 nM for the secretion of IFN-β. Intravenous injection of 3 mg/kg in BALB/c mice could lead to the durable antitumor effect and complete tumor regression through the activation of an adaptive CD8^+^ T cell immune response.

#### SR-717 and MSA-2

In 2020, two back-to-back papers reported two novel non-CDN STING agonists, called SR-717 by the Scripps Research Institute^72^ and MSA-2 by Merck.^73^ SR-717 and MSA-2 were screened by THP-1-based HTS, and they can both bind directly to STING through closed conformation like 2′,3′-cGAMP. SR-717 can bind to STING, with IC_50_ = 7.8 μM competitive with 2′,3′-cGAMP and has an EC_80_ of 3.6 μM for IFN-β induction. Moreover, SR-717 can induce mild *in vivo* antitumor activity such as prolonged survival and facilitate antigen cross-priming with 30 mg/kg administration intraperitoneally. MSA-2 is an orally available non-CDN agonist of human STING, which shows good tolerance in MC38 tumor-bearing C57BL/6 mice. It is administered by a single dose intratumorally (450 μg), subcutaneously (50 mg/kg), and orally (60 mg/kg), leading to the stimulation of IFN-β secretion within tumors, tumor regression, and the development of long-lasting antitumor immunity. Similar to ABZI, MSA-2 binding to STING requires its dimer form, and synthetic covalent MSA-2 dimers are more potent than MSA-2, with EC_50_ as low as 8 ± 7 nM among all the synthesized MSA-2 dimers in terms of secretion of IFN-β in THP-1 cells. Additionally, the cellular potency of MSA-2 undergoes reversible, noncovalent dimerization in solution and is more concentrated because of extracellular acidification that the tumor microenvironment (TME) often owns. These characteristics likely contribute to the favorable efficacy and tolerability observed with effective systemic administration of MSA-2. Notably, both SR-717 and MSA-2 can exhibit a synergistic effect when combined with anti-programmed cell death ligand 1 (PD-L1) therapy through the induction of expression of PD-L1 in an STING-dependent manner, and MSA-2 plus muDX-400 anti-PD1 therapy can achieve 100% survival in advanced MC38 models.

#### G10 series

**Figure 3:**
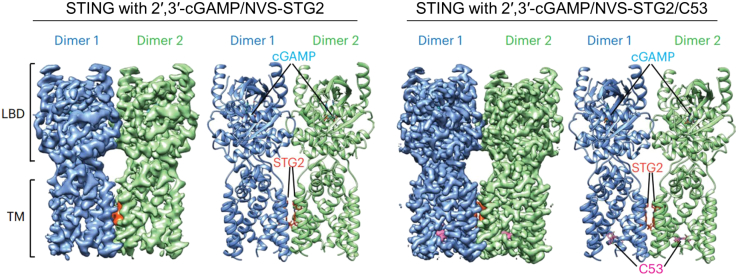
Cryoelectron microscopy density map and atomic model of STING bound to cGAMP/NVS-STG2 or cGAMP/NVS-STG2/C53 The binding sites are located separately at LBD and TMD of STING.^78^

A compound G10 developed in 2015 shows no activation of IRF3 or NF-κB-linked reporters in THP-1, although it can activate the IRF3 activation and IRF3-dependent transcriptional activity in immortalized human fibroblasts.^74^ In 2020, another group tested this G10 in different variants of STING and demonstrated that G10-activated IRF3 is variant dependent, which means that G10 is more potent against the R232 and H232 variants, with EC_50_ = 2.5 and 4.3 μM, respectively, but less active against HAQ carried by THP-1.^75^ This group also investigated the medicinal chemistry of G10-based compound for the improvement of the activation potency, leading to the discovery of one representative compound 53 (C53) with the ability to activate all five major STING variants (human EC_50_ = 185 nM).^76^ One year later, 3,4-dihydroquinazolin2(1H)-one cyclic urea based on G10 was further identified and could activate five major STING variants as well as cynomolgus monkey STING with similar potency.^77^ Interestingly, C53 shows that it binds at a cryptic site in TMD of STING instead of LBD. The binding triggers outward shifts of transmembrane helices in the dimer and induces inter-dimer interactions between these helices to mediate the formation of the high-order oligomer (Figure 3). The concurrent bindings of 2′,3′-cGAMP and C53 to the LBD and the TMD of STING, respectively, promote the formation of higher-order STING oligomers and induce stronger activation of STING than either ligand alone.^79^

#### NVS-STGs

Novartis developed a novel non-CDN STING agonist called the NVS-STGs series based on THP-1-Dual cells from a chemical library containing 250,0000 compounds. Differential scanning fluorimetry supplemented with photoaffinity labeling and quantitative chemical proteomics shows that these NVS-STGs do not interact with the LBD like 2′,3′-cGAMP. Instead, NVS-STGs bind to STING TMD and promote STING oligomerization as a molecular glue.^78^ This study provides another agonist-binding site unlike 2′,3′-cGAMP, as well as their previously reported C53.

#### Other agonists

Ziyuglycoside II, or ST12, is obtained by a cell-based IRF/IFN pathway reporter assay. ST12 is capable of boosting the expression of the NF-κB in THP-1, but it also shows a slight dose dependence in STING^−/−^ cells. The binding affinity revealed by biolayer interferometry shows that the K_d_ of ST12 to human STING^R232^ is 14 μM,^80^ which is far weaker than the reported 3.79 nM for 2′,3′-cGAMP.^41^

One unique chemotype hybridization was adopted to identify novel non-CDN STING agonists. Based on the HTS screening for IRF and NF-κB reporter activity in reconstructed THP-1 cells, a hybrid molecule, pyrazolopyrimidine acid, which combines the motifs of two obtained chemotypes, can also induce micromolar NF-κB and IRF activity in human cellular reporter assays. Structure-activity relationship (SAR) investigation specified the role around the pyrazolopyrimidine and amide linker and offered the optimized STING agonist with overall superior profile *in vitro* and modest abscopal effect *in vivo*.^81^

A different screening platform was achieved by surface plasmon resonance with the immobilization of STING^CTD^ protein to the CM5 sensor chip, followed by an ∼2,000 compound library screening consisting of mostly US Food and Drug Administration (FDA)-approved drugs, which led to the discovery of M335 as a novel non-CDN STING agonist. M335 shows micromolar binding affinity toward STING, activates the STING-TBK1-IRF3 in an STING-dependent manner, and inhibits the growth of multiple refractory cold tumors (MC38, CT26, and B16-F10) administered even intraperitoneally at a 20-mg/kg level.^82^ It is noteworthy that the overall STING activation is only modest compared to 2′,3′-cGAMP.

One group identified KAS-08 derived from previously reported antitumor agent DW2282 with an unknown mechanism as a potent non-CDN STING agonist. KAS-08 has an EC_50_ value of 0.18 μM in the THP-1 ISG LUC reporter assay and shows potency both *in vitro* and *in vivo*.^83^

### Conjugated STING agonists

The conjugation of STING agonists to other chemical/biofunctional moieties such as small molecules, functional groups, and even antibodies is mainly for tackling the instability by ENPP1 and poxin, limited cellular uptake, and short circulatory half-life, achieving sustaining STING activation and optimal antitumor efficacy as well as desired synergistic effects brought by the introduced chemical moieties. Lipids/nanoparticles (NPs)/polymers accompanied by the direct conjugation to STING agonists are also introduced in this chapter.

Common chemical modifications of CDNs occur at nucleobase replacement, ribose substitution, and phosphate modification, as well as internucleotide linkage position. For example, one case reported all possible canonical (3′,3′-linked) CDNs containing an additional two types of nucleobases, cytosine and uracil. However, only 3′,3′-cyclic adenosine-cytidine monophosphate(3′,3′-cACMP) shows a significant but weaker type I IFN response among the synthesized CDNs.^84^ Many novel cyclic adenosine-inosine monophosphate (cAIMP) analogs were designed along with ribose substitution (2′-fluoro/deoxy/hydroxyl), internucleotide linkage position (3′,3′/2′,3′/2′,2′/2′,3′), and phosphorothioate modification. It is surprising that 3′,3′-cAIMP has an EC_50_ of 6.4 μM, in terms of type I IFN induction in human blood *ex vivo*, surpassing 2′,3′-cGAMP (19.6 μM) and 2′,3′-cAIMP (11.2 μM). Further introduction of 2′-fluoro-2′-deoxyribose and phosphorothioate could help the EC_50_ potency to descend to 0.4–4.7 μM, and c-[2′FdAMP(S)-2′FdIMP(S)], with two 2′-fluoro-2′-deoxyriboses and bis-phosphorothioate linkages show the best potency (0.4 μM).^85^ 3′,3′-c-di-IMP has also proven to be a potent STING agonist, slightly greater than c-di-GMP.^86^ For the ribose moiety, some regular modifications are deoxy-fluoro/deoxy/NH_2_/OMe substitutions. It is noteworthy that fluorination seems to have more of an impact on the enzymatic stability and binding affinity of 3′,3′-CDNs than 2′,3′-CDNs since 2′,3′-CDNs generally have greater potency toward human STING (hSTING). 2′-Deoxy modification results in 2′,3′-cyclic deoxyguanosine-deoxyadenosine monophosphate (2′,3′-cdGMP-dAMP), which has comparable potency with 2′,3′-cGAMP, but 2′-NH_2_/OMe modification of 2′,3′-cGAMP ends up with an approximately 10-fold decrease in the type I IFN induction reporter assay.^87^^,^^88^^,^^89^ The locked nucleic acid endo-5′-S-phosphorothioester CDNs were also introduced, and some of them show comparable potency as 2′,3′-cGAMP in the hSTING R232H activation.^89^ As for the phosphate part, thiophosphate is the most widely adopted since it can increase lipophilicity and enzymatic resistance as well as bioactivity. It is worth mentioning that *R* configuration of thiophosphate diastereoisomers always shows greater potency than the corresponding *S* configuration.^90^^,^^91^ Boranophosphate CDNs can also activate the STING signaling pathway in THP-1 LUC reporter cells (EC_50_ <100 and <30 μM in the presence and absence of perfringolysin O, respectively) to the same extent as 2′,3′-cGAMP.^92^ Apart from thiophosphate modification, phosphate backbone replacement by triazole,^93^ thiourea, urea, carbodiimide, and guanidinium^94^ can be found, but unfortunately, there is no report of activity potency. The replacement of phosphodiester linkages by carbamide and thiocarbamide linkages offers an approximately 2-fold increase in the NF-κB pathway activation.^92^

Starting from the development of non-CDN STING agonists, SAR investigations and structural optimizations have become a topic, especially for the ABZI type. Further structural elaborations provide a new potent STING activator possessing EC_50_ values of 0.24 and 39.51 μM for hSTING and murine STING, respectively.^95^ More comprehensive investigations of ABZI led to the identification of CF501,^96^ thieno[2,3-d]imidazole derivatives,^42^^,^^97^ tricyclic scaffold HB3089,^98^ and SHR1032^99^ with a fused tricyclic core but with a mild activation efficacy.^99^

#### Conjugation to small chemical moieties

**Figure 4:**
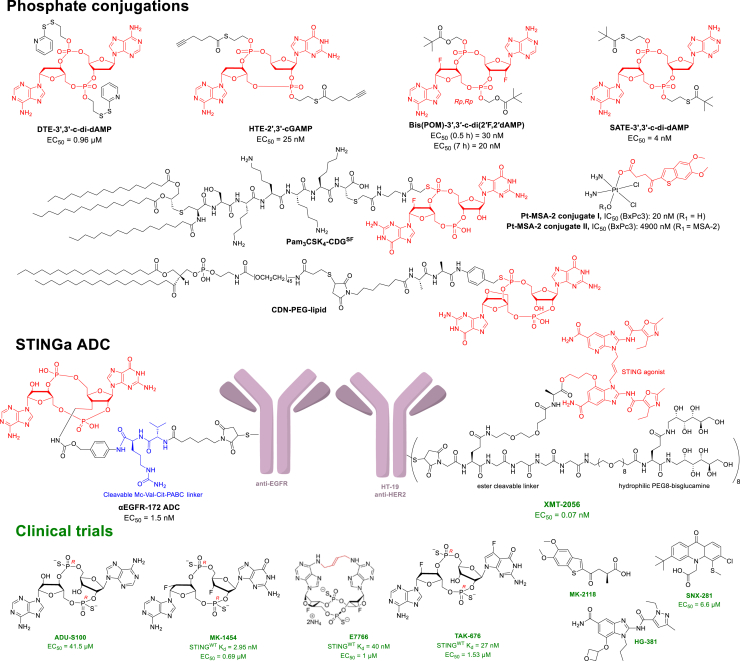
Representative modified/conjugated STING agonists and known structures of STING agonists in clinical trials^85^ Phosphate conjugations utilize prodrug concepts while STINGa ADCs take the place of common cytotoxic payloads such as paclitaxel and mertansine and are conjugated to the antibody through cleavable linkers. Linkers and hydrophilic PEG8-bisglucamine groups play significant roles to desired pharmacokinetic profile and low off-target activity.^100^^,^^101^^,^^102^ STING agonists currently under investigation in clinical trials are highlighted in green.

To overcome the instability and low transmembrane efficiency issues, small chemical moieties are always good choices (Figure 4). To improve the stability and transmembrane ability and boost immune synergies by co-delivery to the same activating polymer drug conjugate (PDC), one group conjugated sulfur- and fluorine-substituted c-di-GMP (CDG^SF^) to a TLR1/2 agonist with three palmitoyls to form novel conjugate Pam_3_CSK_4_-CDG^SF^. The obtaining conjugate could synergistically activate the STING and TLR pathways, better than Pam_3_CSK_4_ or CDG^SF^ alone at 0.1 μM concentration. The *in vivo* results demonstrated that, when co-administered with the antigen ovalbumin, the conjugated adjuvant could significantly inhibit tumor growth with 100% survival at 26 days, while Pam_3_CSK_4_ or CDG^SF^ showed less inhibition and almost no survival at 26 days.^103^

The alkylation of phosphate groups could mask the negative charges and improve the transmembrane efficiency, thereby leading to enhanced potency. For example, 3′,3′-c-di-AMP was modified by reduction-responsive dithioethanol (DTE) to form DTE-3′,3′-c-di-AMP. This conjugate can significantly improve cellular uptake (peak within 2 h, treated with 10 μM) and efficiently be metabolized to its parent 3′,3′-c-di-AMP in THP-1 cells (peak within 6 h), while intracellular levels of 3′,3′-c-di-AMP were almost undetectable when treated with 100 μM 3′,3′-c-di-AMP. As a result, the conjugate has an EC_50_ of 0.96 μM in THP-1-Lucia cells, which is 51-, 43-, and 3-fold more than the 2′,3′-cGAMP, c-di-AMP, and ADU-S100, with EC_50_ values of 48.6, 41.3, and 2.9 μM, respectively.^104^ Furthermore, the introduction of acyloxymethyl or isopropyloxycarbonyl (POC) groups to 3′,3′-c-di(2′-fluoro-2′-deoxy-AMP) could lead to an up to 1,000-fold improvement in potency (EC_50_ = 20 nM) when compared with parent 3′,3′-c-di-AMP,^105^ which should be attributed to both 2′-fluoro and the masking of negative charges. The cellular uptake of bis(POC)-3′,3′-c-di(2′-fluoro-2′-deoxy-AMP) in THP-1 cells peaked within 2 h at micromolar concentrations, while unmodified CDNs could only be slightly detectable at 100× concentration. Moreover, this conjugate has a *T*_1/2_ of 70 min in human plasma, while 2′,3′-cGAMP has a *T*_1/2_ of 30–60 min^106^ Another *S*-acylthioalkyl ester (SATE) group is conjugated to 3′,3′/2′,3′/2′,2′-c-di-dAMP, respectively. Among the three conjugates, SATE-3′,3′-c-di-dAMP has the best EC_50_ value of 5.2 nM, which is comparable to 1.3 nM when transfected with Lipofectamine 2000, indicating its good cell membrane permeability. In addition, SATE-3′,3′-c-di-dAMP has a *T*_1/2_ of 72 h in 20% fetal bovine serum, while 80% 2′,3′-cGAMP was degraded within 72 h. The *in vivo* results show that this conjugate eliminated CT26-Luc tumors at 14 days of intratumoral administration (0.5 mg/kg) and improved the survival up to 60 days, while ADU-S100 had a 75% survival rate at 50 days.^107^ Similarly, 5-hexynoic thioester (HTE) groups were also conjugated to 2′,3′-cdGdAMP to form bis-HTE-dd-cGAMP, thus leading a decrease of EC_50_ in the induction of IFNs from 4.7 μM by dd-2′,3′-cGAMP to 24.6 nM^108^

One classic DNA-damaging agent, cisplatin, was conjugated to STING agonist MSA-2 to form Pt^IV^ -MSA-2 conjugates. Interestingly, conjugate I also exerts unique anticancer mechanisms different from single platinum or MSA-2 because of the axial ligands. The cytotoxic difference in conjugate I and II may be attributed to the cellular uptake difference, and conjugate I has a much higher uptake than conjugate II. Despite a partial decrease in the induction of type I IFNs compared with MSA-2, Pt^IV^-MSA-2 conjugate (I) is highly cytotoxic to Pan02 tumors (IC_50_ = 20 nM) and could effectively suppress tumor growth through the STING agonism rather than the simple mixture of platinum and MSA-2.^109^

#### Conjugation to lipids/NPs/polymers

In terms of these types of conjugations, there are many aspects such as conjugation site, linker, and cargo that should be taken into consideration. It should be pointed out that conjugations are commonly accompanied by the construction of nanosystems. The conjugation site for CDNs usually is a thiophosphate group, a 2′-NH_2_/OH functional group, and an amino group of nucleobases, and the selections depend on the synthetic availability and the minimal impact on the bioactivity. After the emergence of non-CDNs, diABZI and MSA-2 are the most commonly reported STING agonists for the conjugations because of more druggable features. The common conjugation site for diABZI and MSA-2 is the 7-position of the benzimidazole owing to the availability of chemically accessible sites outside of the STING binding pocket and carboxyl group, respectively. For the linkers, cleavable linkers such as cathepsin-cleavable valine-citrulline-*p*-aminobenzyl (PAB) linker and self-immolative linker are common recommendations since the STING activation theoretically requires the release of free STING agonists. The length and the type of linkers are highly correlated with the investigated models and resulting efficacy, which require experimental verifications and optimizations. The selections of which chemical moieties for conjugation are very flexible and some typical examples follow.

A modified CDN was conjugated to thiol-terminated polyethylene glycol-phospholipid (PEGylated lipids) via a dialanine peptide cleavable linker, this CDN-PEG-lipid can further self-assemble into discoid NPs (∼30 nm) and facilitate the formulation into PEGylated lipid nanodiscs (LNDs) (5 mol % loading). The LND-CDN could release free CDNs upon peptidase cleavage in endosomes following cellular uptake, which is several-fold more potent than liposome-CDN and free CDN. Moreover, LND-CDN is more stable in 10% serum and could retain 75% bioactivity over 48 h, while free CDN-PEG-lipid fell to around baseline in 24 h. In animals bearing established MC38 tumors, LND-CDN exhibits a *T*_1/2_ of 12.6 h, which is far better than ∼1 h for Cy5-conjugated cGAMP. Through one single treatment dose on day 7 by intravenous administration, LND-CDNs (5 nmol dose) are able to elicit tumor regression and complete responses in 75% of the animals, while free CDNs or ADU-S100 (100 nmol dose) showed almost no efficacy. The enhanced efficacy by single dose is caused by more effective initial distribution of CDNs to cancer cells and/or DCs throughout the tumor bed. LND-CDNs exhibit high potential in the delivery of CDNs compared with commercial long-circulating PEGylated liposomes.^110^

**Figure 5:**
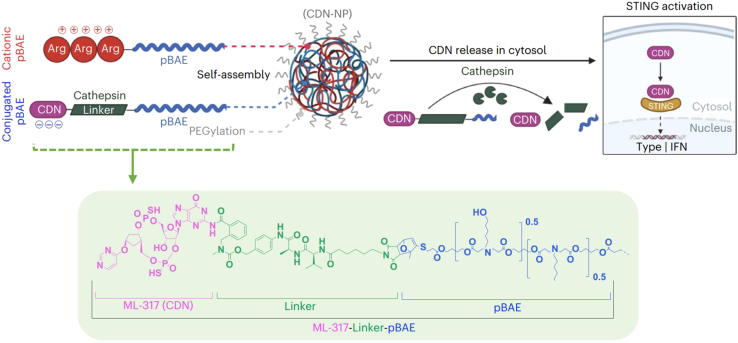
Overview of one representative CDN-conjugated nanoparticle Cationic poly(β-amino ester)s (pBAEs) are created by mixing acrylate-terminated pBAE polymer with arginine oligopeptide, referred to as C6-CR3. Maleimide-modified ML-317 is then conjugated to the pBAE via a Diels-Alder reaction, resulting in the compound known as ML-317-Linker-pBAE. Following CDN conjugation, the ML-317-Linker-pBAE polymer forms electrostatic complexes with the C6-CR3 polymer, leading to the production of covalently conjugated CDN nanoparticles (CDN-NPs). These CDN-NPs undergo PEGylation using NHS-PEG and are subsequently purified and sterilized through filtration. Inside the cell cytoplasm, CDN is released from the CDN-NPs via a cathepsin-cleavable linker.^111^

One modified CDN, ML-347, was covalently conjugated to poly(β-amino ester) NPs through a cathepsin-sensitive linker. The resulting CDN-NPs are stable in mouse plasma for 24 h and could induce higher IRF3 activation in THP-1-Dual cells (EC_50_ = 20.6 nM versus 1146 nM for free CDNs), which might because of an effective internalization by immune cells via caveolin and clathrin endocytosis (90% uptake at 1 nM CDN). In the CT-26 model, CDN-NPs (equal to 0.5 μg CDN dose) could fully reject tumor in 30% of the animals and achieve complete tumor regression in combination with anti-PD-1, expanding the therapeutic window in multiple syngeneic tumor models (Figure 5). More important, this work identified the long-acting role of the conjugate-NPs—in other words, the host cells are mainly responsible for primary tumor clearance, whereas cancer cells stock the NPs that are released over time to activate nearby immune cells.^111^ STING agonist MSA-2 was also conjugated to sonodynamic semiconducting polymer core assembled from a semiconducting polymeric nanoagonist (SPNM) via a ^1^O_2_-cleavable linker called EOPD. The singlet oxygen can be produced from SPNM upon sono-irradiation activation, leading to the release of caged MSA-2 (elution peak at 9.7 min upon 10-min sono-irradiation). Despite that SPNM-mediated IFN-β secretion by DCs could reach ∼80% effect in comparison with MSA-2, SPNM-mediated STING activation could achieve 83.5% inhibition for primary tumors and 76.4% for distant tumors, while MSA-2 retained only 37.2% and 51.9%, respectively. The median survival of the free MSA-2 group was 16 days, and the SPNM group showed 100% survival within 32 days. In principle, the SPNM group could promote the effector T cell infiltration and long-term immunological memory.^112^

**Figure 6:**
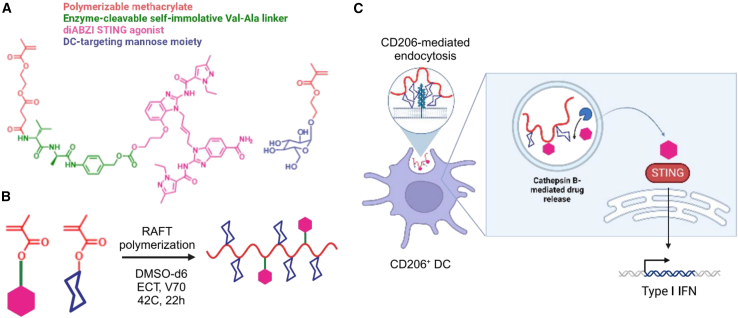
A polySTING polymeric prodrug (A) Structures of two basic monomers: one is the conjugated STING agonist that is the enzyme-cleavable methacrylate-based diABZI STING agonist and the other is mannose ethyl methacrylate. (B) The polymerization induced by reversible addition-fragmentation chain transfer (RAFT) helps in the formation of polySTING. (C) polySTING targets CD206^+^/mannose receptor^+^ professional antigen-presenting cells, leading to endosomal prodrug cleavage and agonist release and STING activation in dendritic cells. Adapted from Nguyen et al.^113^

PolySTING (∼12 kDa), a DC-targeted polymeric prodrug platform, is made up the polymerization of STING agonist diABZI monomer and mannose ethyl methacrylate monomer. Non-CDN diABZI was conjugated to polymerizable methacrylate through an enzyme-cleavable self-immolative Val-Ala linker (Figure 6). PolySTING could be internalized by receptor-mediated endocytosis in CD206^+^ APCs, and the free diABZI could be released after endosomal cathepsin cleavage. In the tumor-draining lymph nodes, polySTING could produce a 9.3-fold increase in IFNβ1 expression and a 13-fold increase in CXCL10 expression compared to free diABZI. In B16-F10 tumor-bearing C57BL/6 mice, polySTING significantly inhibited tumor growth over freeform and prolonged survival to 22 days with minimal side effects compared with 11 days for the free STING group.^113^

HA-MSA2, formed by the conjugation between hyaluronic acid (HA) and STING agonist MSA-2, was investigated for the treatment of aggressive brain tumor glioblastoma (GBM). This conjugate could achieve ∼2-fold higher stimulation of STING signaling than free MSA-2. In the syngeneic GBM mouse model, HA-MSA2 conjugate (5 μg/mouse) could delay tumor growth and prolong survival up to 25 days through convection-enhanced delivery, while free MSA-2 showed 20 days of median survival. The enhanced efficacy is caused by remodeling the tumor immune microenvironment with increased filtration of immune cells.^114^

**Figure 7:**
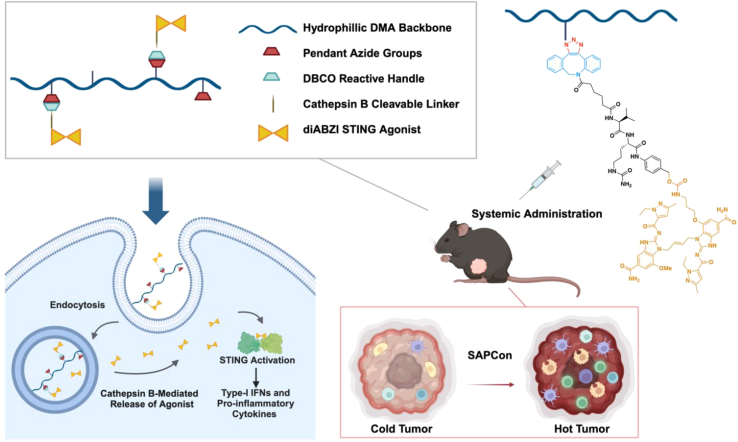
Overview of STING-activating polymer drug conjugates The STING-activating polymer drug conjugates (SAPCon) platform comprises a hydrophilic poly(dimethylacrylamide) (DMA) backbone copolymerized with azide-functionalized monomers, enabling strain-promoted azide-alkyne cycloaddition to a dibenzocyclooctyne(DBCO)-functionalized, cathepsin B-cleavable diABZI STING agonist. Designed for intravenous delivery, SAPCon enhances tumor-targeted accumulation and cellular internalization. Upon reaching the tumor microenvironment, tumor-associated myeloid cells enzymatically cleave the platform to release diABZI, thereby amplifying STING activation within tumor tissue. This mechanism drives the reprogramming of the tumor-immune microenvironment, potentiating antitumor immune responses. Adapted from Sheehy et al.^117^

A potent nanoradiosensitizer called GA-MOF was synthesized by conjugating STING agonist 2′,3′-cGAMP on metal-organic frameworks (MOFs) for synergistic radiosensitization and STING activation. This MOF conjugation improved the cellular uptake of 2′,3′-cGAMP by 34.5-fold by confocal laser scanning microscopy, showed a 3-fold increase (2.34 μM versus 6.98 μM for 2′,3′-cGAMP) as for IRF response, and prolonged plasma retention (the area under the receiver operating characteristic curve is 1.48-fold higher than free 2′,3′-cGAMP). In subcutaneous MC38, CT26, Panc02, and SCC7 models, GA-MOF could increase the average tumor growth inhibition from 53% to 64% for 2′,3′-cGAMP to 71%–90% by creating immune cell-rich nodules and converting them into immunostimulatory hotspots in conjunction with radiotherapy.^115^ A similar platform was also constructed by conjugating 2′,3′-cGAMP to a two-dimensional nanoscale metal-organic layer (MOL) for simultaneous STING activation and radiosensitization. The MOL conjugation facilitated higher cellular uptake and longer intratumoral retention (54-fold higher fluorescence in 24 h) and enhanced STING activation in terms of IRF response (EC_50_ = 103 nM versus 665 nM for free 2′,3′-cGAMP). In CT26 and MC38 tumor models, cGAMP/MOL treatment inhibited tumor growth to 99.7% and 96.4%, respectively, while cGAMP inhibited tumor growth to 88% and 64.7%, respectively. Notably, the MOL can also sustain cGAMP in tumors for prolonged STING activation.^116^ Another type of polymer-drug conjugate, SAPCon, consisted of diABZI STING prodrug and hydrophilic poly(dimethylacrylamide-co-azido-ethylmethacrylate) polymer chains through a cathepsin B-responsive linker (Figure 7). The EC_50_ activity of SAPCon[100 kDa] for IRF3 response is 24.1 nM compared to 1.3 nM for free diABZI. In C57BL/6 mice with EO771 mammary tumors, SAPCon[100 kDa] (0.009 μmol diABZI, 0.68 mg/kg) could achieve a 37.5% complete response rate, while free diABZI showed almost no efficacy.^117^ In addition to the innate immune response, the systemic antitumor response can be trigged and elicited among these platforms, including cGAMP/MOL, GA-MOF, and SAPCon, in combination with the anti-PD-1/PD-L1 immune checkpoint inhibitor.

#### STING agonist ADC

To date, the FDA has approved 11 antibody-drug conjugates (ADCs), such as gemtuzumab ozogamicin (Mylotarg), for the treatment of CD33^+^ acute myeloid leukemia and trastuzumab-SMCC-DM1 for the treatment of HER2^+^ metastatic breast cancer.^118^ The general concept has been prevalent with the combination of cytotoxic payloads with tumor-specific targets through designed linkers since the first FDA approval of gemtuzumab ozogamicin.^119^ Similar to ADC, general factors should be taken into consideration to form STING agonist ADC (STINGa ADC) (Figure 4), such as payload structural optimization for suitable conjugation, linker selection, and the scaffold optimization. Similarly, adopting non-CDN STING agonists seems to be a trend because of manufacturing accessibility and more druggable features in pharmacokinetics and pharmacodynamics (generally optimized for the best profile of activity and hydrophilicity). For the linkers, cleavable linkers such as cathepsin-cleavable linker, self-immolative linker, and non-cleavable linkers such as maleic amide have been investigated. It turns out that the cleavable linkers could be the most suitable selections because they are more efficient in releasing free drug without additional chemical moiety. As for the scaffold, hydrophilic motifs, self-hydrolyzing maleimide motif, a branching point that positions the hydrophilic group adjacent to the payload and places the payload closer to the antibody, are some important factors to consider to improve related stability, activity, and tolerability.^100^

In 2022, an α-epidermal growth factor receptor-172 (αEGFR-172) ADC was generated by conjugating cGAMP analog IMSA172 modified by one aminoethyl group at the 3′-position of guanosine to murine-anti-human EGFR antibody (mu-αEGFR) through a self-immolative Mc-Val-CitPABC linker. The obtaining STINGa ADC can stimulate potent IFN response with an EC_50_ of 1.5 nM compared to 35 μM for IMSA172. The systemic administration of the αEGFR-172 (200 μg each time, intraperitoneal injection three times) can be well tolerated and achieve tumor remission in 60% of mice bearing B16-F10 tumors, while anti-PD-L1 antibody (200 μg) only modestly reduced tumor growth. However, the combination of αEGFR-172 with anti-PD-L1 antibody completely suppressed tumor growth and led to the survival of all mice. IMSA172 has good stability within 24 h in human sera, while only 10% cGAMP was left within 24 h. Most important, αEGFR-172 can maintain half of the peak level (4 h after injection) up to 12 days in mouse models.^101^

Typical examples of STINGa ADCs were mainly screened, optimized, and developed by Mersana Therapeutics based on their Immunosynthen platform with the introduction of physicochemical and drug-like properties since the first report in 2019.^101^^,^^120^^,^^121^ For the payload, non-CDN STING agonist diABZI was selected, with subtle modifications evaluated by activity and hydrophilicity. Both cleavable and non-cleavable linkers were screened to determine the optimal choice for the delivery, and an ester cleavable linker was prioritized. The scaffold was finally optimized to HT-19, a HER2-targeted antibody specifically developed for ADC. Further incorporation of PEG8-bisglucamine scaffold was demonstrated to be the lead platform screened by the *in vivo* activity, pharmacokinetic profile, and tolerability.^100^ The Immunosynthen platform resulted in the production of XMT-2056, which demonstrated excellent tolerance in non-human primates at exposure levels that were significantly higher than those needed for antitumor activity,^122^ improving antitumor activity in a ratHER2-engineered EMT-6 syngeneic mouse model in combination with anti-PD1 therapy.^123^ Antibody-dependent cell mediated cytotoxicity was also observed accompanied by the STING activation, leading to the Fcγ receptor (FcγR)-mediated internalization in FcγRI-expressing myeloid cells^124^ and improvement of cancer cell-killing activity of FcγRIII^+^ (CD16^+^) immune cells.^125^ Both cancer cells and myeloid cells contributed to the ultimate antitumor activity, and the induction of type III IFN in TME tumor cells in turn regulated type I IFN and other cytokines/chemokines required for robust anticancer response.^102^ As a result, in 2022, XMT-2056 (drug-to-antibody ratio = 8) was granted an orphan drug designation for use as a potential therapeutic option for patients with gastric cancer.

#### Other conjugations

There is one classification of conjugated STING agonist as probes. For example, one photoaffinity probe of 2′,3′-cGAMP was designed to capture and isolate 2′,3′-cGAMP-binding proteins. The conjugation happens at the 6-adenosine position and contains a terminal alkyne and diazirine group for photocrosslinking. Following photocrosslinking, bound proteins are covalently crosslinked to 2′,3′-cGAMP probes and can be conjugated via click chemistry to fluorescence tag (Rh-N_3_) or to affinity tag (biotin-N_3_). As a result, EF1A1 is finally identified as associating with 2′,3′-cGAMP.^126^ Moreover, fluorescent/biotinylated conjugation of STING agonist (2′,3′-cGAMP-biotin conjugate, 2′,3′-cGAMP-Cy5 conjugate) is also common for the identification of correlated proteins such as ASFV B175L^127^ or direct monitoring of 2′,3′-cGAMP.^128^

The component for proteolysis-targeting chimera is generally the inhibitor of the protein of interest. However, one group adopted STING agonist diABZI for the conjugation to von Hippel-Lindau to promote STING degradation and suppress STING-mediated innate immune activation. The conjugation site is the amine group of diABZI, which has less impact on the STING binding. For the linkers, a C8-linked conjugate could achieve a 92% STING degradation with a half-maximal degradation concentration of 0.227 μM in Caki-1 cells.^129^

### STING agonist in clinical trials

General CDN-type STING agonists require intratumoral injection; have rapid clearance from the body, poor membrane permeability, and a narrow therapeutic window; and are prone to induce systemic immunotoxicity (e.g., high IFN-β levels). The first clinical trials of STING agonists were initiated around a decade ago, after the emergence of ADU-S100 (Table 1),^5^ and CDN-type STING agonists take up about half of all the clinical trials. Despite many clinical trials not being able to disclose the structures for reasons of confidentiality, the clinical trials with known structures all adopted suitable modifications to overcome the inherent disadvantages of CDNs and potentiate efficacy. For example, all of the tested CDNs (ADU-S100, MK-1454, TAK-676) are 2′,3′-linked and contain two phosphorothioate linkages with *R* configurations. Both MK-1454 and TAK-676 contain two fluorinated modifications. E7766 is a macrocycle-bridged STING agonist inspired by the U-shaped conformation upon binding to STING dimer proteins. E7766 also contains two phosphorothioate linkages (*R*p*R*p isomer) and two fluorine atoms in the sugars.

**Table 1:** STING agonists in clinical trials

Name	Developer	Phase	NCT no.	Indication/route	Combination	Notes
ADU-S100 (MIW815)	Novartis	1∗	NCT03172936 (2017–2022)	solid tumors and melanoma (IT)	spartalizumab	minimal antitumor response
MK-1454	Merck	1∗∗	NCT03010176 (2017–2024)	advanced/metastatic solid tumors or lymphomas (IT)	pembrolizumab	24% SR with pembrolizumab
E7766	Eisai	1∗	NCT04144140 (2019–2023)	advanced solid tumors or lymphomas (IV)	–	ongoing
SB 11285	Spring Bank/ImmunoVir	1∗∗	NCT04096638 (2019–2024)	advanced solid tumors (IV)	atezolizumab	good tolerance, efficacy ongoing
IMSA101	ImmuneSensor	1/2∗∗	NCT04020185 (2019–2024)	advanced treatment-refractory malignancies (IT)	checkpoint inhibitors, chemotherapy	ongoing
GSK3745417	GlaxoSmithKline	1∗∗∗	NCT03843359 (2019–)	refractory/relapsed solid tumors	dostarlimab	ongoing
BMS-986301	Bristol Myers Squibb	1∗∗	NCT03956680 (2019–2024)	advanced solid tumors (IT/IV/IM)	nivolumab, ipilimumab	ongoing, early data pending
TAK-676	Takeda	1∗∗	NCT04879849 (2021–2024)	NSCLC, TNBC, and HNSCC (IV)	pembrolizumab + radiotherapy	enrollment in expansion cohorts
SNX281	Stingthera	1∗	NCT04609579 (2020–2024)	advanced solid tumors and lymphoma (IV)	pembrolizumab	ongoing
HG-381	HitGen	1∗∗∗∗	NCT04998422 (2021-)	advanced solid tumors (IV)	–	ongoing
CDK-002 (exoSTING)	Codiak	1∗∗	NCT04592484 (2020–2022)	TNBC, HNSCC, ATC, and cSCC (IV)	–	CDNs encapsulated by exosome
MK-2118	Merck & Dohme	1∗	NCT03249792 (2017–2023)	advanced/metastatic solid tumors or lymphomas (IT)	pembrolizumab	manageable toxicity, limited antitumor activity
CRD3874-SI	University of Maryland	1∗∗∗∗	NCT06626633 (2024–)	relapsed/refractory acute myeloid leukemia (IV)	–	ongoing
XMT-2056	Mersana Therapeutics	1∗∗∗∗∗	NCT05514717 (2023–)	advanced/recurrent solid tumors that express HER2 (IV)	–	on hold because of grade 5 SAE

Phase status: ∗terminated; ∗∗completed; ∗∗∗active, not recruiting; ∗∗∗∗recruiting; ∗∗∗∗∗pending. ATC, anaplastic thyroid carcinoma; cSCC, cutaneous squamous cell carcinoma; HNSCC, head and neck squamous cell carcinoma; IM, intramuscular route; IT, intratumoral route; IV, intravenous route; NSCLC, non-small cell lung cancer; SAE, side adverse effect; SCCHN, squamous cell carcinoma of the head and neck; SR, sustainable remission; TNBC, triple-negative breast cancer.

Many of the clinical trials have already been terminated. For example, phase 1 clinical trials of ADU-S100 (MIW815), a modified first-generation CDN structure, showed good tolerance even in patients with anti-PD-1 refractory disease, but the antitumor responses were minimal.^130^ MK-2118 is an MSA-2 type STING agonist. Although preclinical data have shown promise, MK-2118 ± pembrolizumab and subcutaneous MK-2118 + pembrolizumab have manageable toxicity but limited antitumor activity in phase 1 clinical trials, with objective responses below 6%.^131^^,^^132^^,^^133^

Among the completed phase 1 trials, MK-1454 alone does not show the expected efficacy, but it does show sustainable remission in combination with PD-1 antibody pembrolizumab in 24% of the patients.^134^ The encouraging efficacy and an acceptable safety profile support the phase 2 trials for the continued development of the combination treatment. SB 11285 can be administrated intravenously and shows good tolerance with or without atezolizumab in phase 1 trials; further efficacy results are still under evaluation.^135^^,^^136^ TAK-676 alone or plus pembrolizumab has a manageable safety profile, encouraging early clinical responses, and durable stable disease, along with robust pharmacodynamic modulation consistent with STING agonism. Enrollment in expansion cohorts is ongoing.^137^^,^^138^^,^^139^ Despite a lack of detailed clinical data, BMS-986301, as a CDN analog, has shown potential to activate the STING pathway effectively, resulting in enhanced antitumor immunity in phase 1 trials.^140^ Clinical trials of SNX-281,^141^ GSK3745417,^142^ E-7766,^143^ HG-381, and CRD3874-SI^144^^,^^145^ are ongoing.

STINGa ADC XMT-2056 can specifically deliver the agonist into a tumor site and has shown quite promising preclinical results. However, FDA placed a clinical hold on this phase 1 trial because a grade 5 side adverse effect (SAE) occurred in the dose-escalation portion of the trial, which was deemed to be correlated with XMT-2056; the cause of death is still under investigation.

## Conclusion and Perspectives

Nature has chosen many types of STING agonists directly or indirectly to prevent cells from being infected. The activation of STING pathway, a crucial component of the innate immune system, leads to the release of type I IFNs and proinflammatory cytokines, thereby inducing the adaptive immune response and giving DCs and macrophages the ability to present antigens and activating T cells. CDNs, as natural STING agonists, are confronted with many obstacles against the druggable development of cancer immunotherapy, with either single or combined administration in clinical trials, including but not limited to inherent structural defects, topical administration, fast clearance, narrow therapeutic window, high risk of systemic off-target toxicity, TME adaptive resistance, and limited clinical efficacy.

The current solution strategies and future directions could focus on the following aspects: the development of second-generation non-CDN STING agonists such as SR-717, MSA-2, and diABZI, along with druggable investigations, including pharmacodynamics and SAR explorations as well as pharmacokinetic optimizations can tackle with low membrane permeability, instability issues and provide other administration routes. To overcome the off-target toxicity and better deliver STING agonists into tumor sites, nanosystems are widely reviewed^25^^,^^146^^,^^147^^,^^148^^,^^149^ and common advantages can be achieved such as improved pharmacokinetic behavior, reduced side effects, and low dosing administration. However, NP-aided delivery of STING agonists still faces several hurdles such as potential toxicity and side effects, circulation-affected instability of the nanosystem, and, most important, clinical support.

Considering that all of the clinical trials of STING agonists are from certain modifications or conjugations except exoSTING, the conjugations might hold promise to fully potentiate and develop STING agonist-based cancer immunotherapy with good tolerance and expected efficacy. Despite that the direct conjugations of both CDN STING agonists and non-CDN STING agonists supplemented with suitable modifications to several chemical moieties and polymers as well as antibodies have shown promising preclinical results, only a few conjugates such as STINGa ADC have entered phase 1 clinical trials. The conjugated items to STING agonists along with druggable investigations can be further expanded and developed for better targeted and effective delivery. Of course, the combination with immune PD-1/PD-L1 antibody and other therapies have already been investigated in clinical trials and might also show synergistic efficacy.

Unfortunately, previous clinical studies of synthetic STING agonists offer overall disappointing outcomes. To evade the immune system for cancer cells, aberrant cytoplasmic dsDNA accumulation and the immunosuppression of cGAS-STING signaling are beneficial and frequently observed; this is likely to be related to immune checkpoint blockade resistance. Still, the comprehensive elucidation of the molecular mechanisms governing tumor responsiveness to STING agonism, coupled with precise patient stratification strategies, remain critical for optimizing therapeutic efficacy. Current challenges stem from the absence of reliable predictive biomarkers—whether genomic alterations (e.g., STING haplotype variations), transcriptional signatures of tumor-immune interactions, or differential sensitivity across cancer subtypes to identify the patient populations most likely to benefit.^150^ This knowledge gap has contributed to suboptimal clinical outcomes in trials evaluating synthetic STING agonists, underscoring the urgent need for mechanistic studies to decode context-dependent pathway activation and resistance mechanisms within heterogeneous TMEs.

Notably, the treatment of STING agonists to STING agonism has proven difficult for those cell lines without a suitable expression of STING, which is due to epigenetic silencing of the promoter regions of cGAS-STING by DNA hypermethylation.^151^ Therefore, some possible interventions stimulating the tumoral cGAS-STING pathway by utilizing cytotoxic chemotherapies such as DNA-damaging agents^152^ to increase cytoplasmic DNA fragments or molecular-targeted drugs such as epigenetic inhibitors^153^ to restore cGAS-STING signaling have shown promise in preclinical studies. The development of reliable predictive biomarkers and the restoration of the cGAS-STING pathway, together with deeper mechanistic studies, might finally produce clinical success in STING-based immunotherapy in the future.

## Acknowledgments

This work was supported by the 2024 Henan Provincial Key Research and Development and Promotion Special Project (Key Science and Technology) (grant no. 242102110050) and the new round of key disciplines in Henan Province - Veterinary Medicine (discipline no.: 312).

## Author contributions

S.Q.: conceptualization, writing – review & editing, and resources. H.D.: conceptualization, supervision, writing – original draft analysis, writing – review & editing, and resources.

## Declaration of interests

The authors declare no competing interests.

## Declaration of generative AI and AI-assisted technologies in the writing process

During the preparation of this work, the authors used DeepSeek (https://chat.deepseek.com/) to polish the language. After using this tool/service, the authors reviewed and edited the content as needed and take full responsibility for the content of the publication.

## Supplemental information

Document S1. Table S1

Document S2. Article plus supplemental information
