# Supplementary material for: Conjugated STING agonists
Source: Mol Ther Nucleic Acids. 2025 Mar 31;36(2):102530. doi: 10.1016/j.omtn.2025.102530 (PMC12032345; doi:10.1016/j.omtn.2025.102530)
Supplement: Document S1. Table S1 [file mmc1.pdf]

OMTN, Volume *36*

Supplemental information

Conjugated STING agonists

Shuhao Qu and Hong Dai

Table S1. Non-CDNs STING agonists in preclinical studies

Name	Binding affinity	EC_{50}	Structure	In vivo	Reference
Dispiro diketopiperzine (DSDP)	/	> 20 μM (ISG54 promoter)	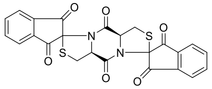	/	1
BNBC	/	1.2 μM (ISG54 promoter)	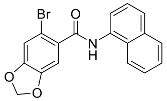	/	2
diABZI	$K_d^{app} \approx 1.6$ nM (Chemoproteomic analysis)	$STING^{WT}$, 130 nM $STING^{HAQ}$, 190 nM $STING^{R232H}$, 200 nM	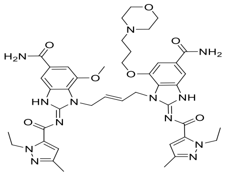	1.5 mg/kg, i.v., three injections, > 90% TGI Tumor free in 80% mice	3
M335 (Fendiline)	~10 μM (SPR)	> 20 μM	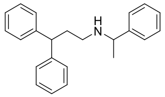	300 μg, every two days, i.t., 82% TGI	4
α-Mangostin	137 μM (MST)	> 25 μM	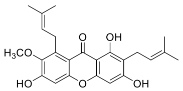	/	5
C11	Not detected (fluorescence-based thermal shift)	> 25 μM	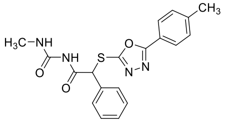	/	6

Ziyuglycoside II (ST12)	14 μM (BLI)	> 10 μM	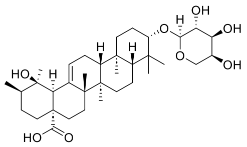	/	7
KAS-08	/	0.33 μM	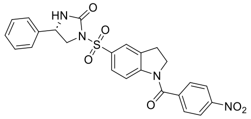	15 mg/kg, i.v. + cGAMP (2 μg, i.t.), delay tumor growth	8
NVS-STG2	/	5.2 μM	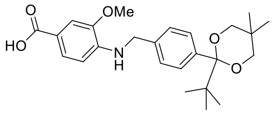	800 μg, i.t., three injections, slow tumor growth	9
C53	/	185 nM (IRF3 reporter)	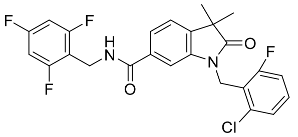	/	10,11
SR-717	/	2.1 μM	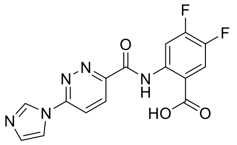	30 mg/kg, I.P., seven injections, >90% TGI	12
MSA-2	Not detected (monomer) Nanomolar (dimer)	8.3 μM	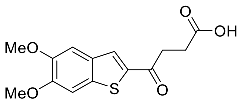	30 mg/kg, i.p.; 60 mg/kg, orally, single dose; complete tumor regression in 80%-100% mice	13
Chemotype Hybridization	/	IRF, 1.4 μM NF-κB, 1.8 μM	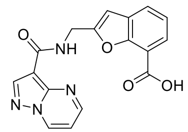	30 μg, i.t., 2 injections 100% TGI, 50% tumor free in injected tumors; 40% TGI in distal tumor	14

MST, microscale thermophoresis; BLI, bio-layer interferometry; i.t., intratumorally; i.v., intravenously; i.p., intraperitoneally; TGI, tumor growth inhibition.

References

1. Liu, B., Tang, L., Zhang, X., Ma, J., Sehgal, M., Cheng, J., Zhang, X., Zhou, Y., Du, Y., Kulp, J., et al. (2017). A cell-based high throughput screening assay for the discovery of cGAS-STING pathway agonists. Antiviral Res.147:37-46.
2. Zhang, X., Liu, B., Tang, L., Su, Q., Hwang, N., Sehgal, M., Cheng, J., Ma, J., Zhang, X., Tan, Y., et al. (2019). Discovery and Mechanistic Study of a Novel Human-Stimulator-of-Interferon-Genes Agonist. ACS Infect Dis.5(7):1139-1149.
3. Ramanjulu, J. M., Pesiridis, G. S., Yang, J., Concha, N., Singhaus, R., Zhang, S.-Y., Tran, J.-L., Moore, P., Lehmann, S., Eberl, H. C., et al. (2018). Design of amidobenzimidazole STING receptor agonists with systemic activity. Nature.564(7736):439-443.
4. Zhao, M., Fan, W., Wang, Y., Qiang, P., Zheng, Z., Shan, H., Zhang, M., Liu, P., Wang, Y., Li, G., et al. (2024). M335, a novel small-molecule STING agonist activates the immune response and exerts antitumor effects. Eur J Med Chem.264:116018.
5. Zhang, Y., Sun, Z., Pei, J., Luo, Q., Zeng, X., Li, Q., Yang, Z., Quan, J. (2018). Identification of alpha-Mangostin as an Agonist of Human STING. ChemMedChem.13(19):2057-2064.
6. Gall, B., Pryke, K., Abraham, J., Mizuno, N., Botto, S., Sali, T. M., Broeckel, R., Haese, N., Nilsen, A., Placzek, A., et al. (2018). Emerging Alphaviruses Are Sensitive to Cellular States Induced by a Novel Small-Molecule Agonist of the STING Pathway. J Virol.92(6).
7. Cui, X. L., Xie, Y. L., Zhang, M., Gao, J. K., Zhou, X. J., Ding, J. W., Cen, S., Zhou, J. M. (2022). Identification of Ziyuglycoside II from a Natural Products Library as a STING Agonist. Chemmedchem.17(11).
8. Jung, H. R., Jo, S., Jeon, M. J., Lee, H., Chu, Y., Lee, J., Kim, E., Song, G. Y., Jung, C., Kim, H., et al. (2022). Development of Small-Molecule STING Activators for Cancer Immunotherapy. Biomedicines.10(1).
9. Li, J., Canham, S. M., Wu, H., Henault, M., Chen, L., Liu, G., Chen, Y., Yu, G., Miller, H. R., Hornak, V., et al. (2024). Activation of human STING by a molecular glue-like compound. Nat Chem Biol.20(3):365-372.
10. Pryde, D. C., Middya, S., Banerjee, M., Shrivastava, R., Basu, S., Ghosh, R., Yadav, D. B., Surya, A. (2021). The discovery of potent small molecule activators of human STING. Eur J Med Chem.209:112869.
11. Lu, D., Shang, G., Li, J., Lu, Y., Bai, X.-c., Zhang, X. (2022). Activation of STING by targeting a pocket in the transmembrane domain. Nature.604(7906):557-562.
12. Chin, E. N., Yu, C. G., Vartabedian, V. F., Jia, Y., Kumar, M., Gamo, A. M., Vernier, W., Ali, S. H., Kissai, M., Lazar, D. C., et al. (2020). Antitumor activity of a systemic STING-activating non-nucleotide cGAMP mimetic. Science.369(6506):993-999.
13. Pan, B. S., Perera, S. A., Piesvaux, J. A., Presland, J. P., Schroeder, G. K., Cumming, J. N., Trotter, B. W., Altman, M. D., Buevich, A. V., Cash, B., et al. (2020). An orally available non-nucleotide STING agonist with antitumor activity. Science.369(6506):935-945.
14. Cherney, E. C., Zhang, L. P., Lo, J. L., Huynh, T., Wei, D., Ahuja, V., Quesnelle, C., Schieven, G. L., Futran, A., Locke, G. A., et al. (2022). Discovery of Non-Nucleotide Small-Molecule STING Agonists via Chemotype Hybridization. J Med Chem.65(4):3518-3538.
